# Maternal Influences on the Transmission of Leukocyte Gene Expression Profiles in Population Samples from Brisbane, Australia

**DOI:** 10.1371/journal.pone.0014479

**Published:** 2010-12-31

**Authors:** Elizabeth Mason, Graham Tronc, Katia Nones, Nick Matigian, Jinhee Kim, Bruce J. Aronow, Russell D. Wolfinger, Christine Wells, Greg Gibson

**Affiliations:** 1 School of Biological Sciences, University of Queensland, St. Lucia, Australia; 2 Wesley Medical Centre, Auchenflower, Australia; 3 Eskitis Institute for Cell and Molecular Therapies, Griffith University, Nathan, Australia; 4 School of Biology, Georgia Institute of Technology, Atlanta, Georgia, United States of America; 5 Computational Medicine Center, Cincinnati Children's Hospital Medical Center, Cincinnati, Ohio, United States of America; 6 SAS Institute Inc., Cary, North Carolina, United States of America; New York University, United States of America

## Abstract

Two gene expression profiling studies designed to identify maternal influences on development of the neonate immune system and to address the population structure of the leukocyte transcriptome were carried out in Brisbane, Australia. In the first study, a comparison of 19 leukocyte samples obtained from mothers in the last three weeks of pregnancy with 37 umbilical cord blood samples documented differential expression of 7,382 probes at a false discovery rate of 1%, representing approximately half of the expressed transcriptome. An even larger component of the variation involving 8,432 probes, notably enriched for Vitamin E and methotrexate-responsive genes, distinguished two sets of individuals, with perfect transmission of the two profile types between each of 16 mother-child pairs in the study. A minor profile of variation was found to distinguish the gene expression profiles of obese mothers and children of gestational diabetic mothers from those of children born to obese mothers. The second study was of adult leukocyte profiles from a cross-section of Red Cross blood donors sampled throughout Brisbane. The first two axes in this study are related to the third and fourth axes of variation in the first study and also reflect variation in the abundance of *CD4* and *CD8* transcripts. One of the profiles associated with the third axis is largely excluded from samples from the central portion of the city. Despite enrichment of insulin signaling and aspects of central metabolism among the differentially expressed genes, there was little correlation between leukocyte expression profiles and body mass index overall. Our data is consistent with the notion that maternal health and cytokine milieu directly impact gene expression in fetal tissues, but that there is likely to be a complex interplay between cultural, genetic, and other environmental factors in the programming of gene expression in leukocytes of newborn children.

## Introduction

A growing body of evidence points toward a fetal origin for influences on chronic disease susceptibility, in part reflecting transmission of maternal health effects [1] Most apparent for conditions such as Fetal Alcohol Syndrome, maternal influences are now also implicated in childhood obesity and early onset Type 2 Diabetes. Barker's thrifty phenotype hypothesis [2]–[3] argues that genetic reprogramming occurs in fetuses developing in undernourished mothers, preparing them for rapid assimilation of nutrients that turns out to be inappropriate when food is actually readily available. On the other hand, obese mothers tend to give birth to overweight children, even after adjusting for genetic correlations [4]. The mechanism of any genetic reprogramming is unknown, but seems likely to involve epigenetic modification of the chromatin [5]–[7] and to be reflected in changes in gene expression profiles that should be observable already in newborn babies.

Umbilical cord blood is an appropriate tissue to look for such effects, since it is readily obtained at birth and can be sampled from populations. A study in Thailand, for example, detected the influence of maternal exposure to arsenic in altered profiles of cord blood gene expression [8]. Despite the extensive literature on the development of immune competence in babies and its relevance to cord blood transplantation [9]–[11], profiling of leukocyte gene expression in newborns is in its infancy. A recent study of 30 mother-child pairs from the Czech Republic reported 2-fold differential expression of 841 genes involved in multiple immunity-related pathways [12]. Likewise, Jiang et al [13]–[14] have also documented up-regulation of interferon and interleukin signaling in maternal relative to cord blood monocytes and dendritic cell samples from small numbers of donors; these studies also described differences in immune competence assessed by LPS challenge. Differences in adult peripheral blood expression have also been documented between mothers who did or did not transmit HIV to their children [15], but there is a dearth of information on the population structure of maternal and neonate gene expression profiles.

The advent of genomic profiling tools has nevertheless enabled systematic evaluation of the genetic influences on the expression of individual genes in adult peripheral blood monocytes. Two notable studies involving approximately 1,000 participants in Icelandic [16] and Mexican American cohorts [17] have documented correlations between gene expression and obesity as well as serum cholesterol levels, respectively. Our own studies in Morocco [18]–[19] have established that over a third of the leukocyte transcriptome is altered in adult samples from nomads, rural villagers, and urban residents, mediated in part by a complex influence of gender-specific cultural practices. We also found that genetic regulatory influences are robust to the environmental sources of variance such that regulatory polymorphisms exert the same influence on transcript abundance across locations and in both Arab and Berber ethnicities [19]. Others have also documented long-term environmental/cultural influences on adult gene expression: for example, low levels of early-life stress correlated with socioeconomic status are reflected in exaggerated inflammatory response fifty years later, inferred from peripheral blood monocyte gene expression profiles [20].

In this study, we extend the description of the population structure of leukocyte gene expression profiles to a large cosmopolitan city, including maternal and new-born cord blood samples, to address three questions: (i) is there evidence of maternal transmission of profiles to their babies, (ii) does any such transmission differ between obese, normal weight, and gestational diabetic mothers, and (iii) how are these influences distributed across regions of the city? We conducted two sequential studies in Brisbane (Queensland, Australia), a modern city of 2 million people with marked socio-economic differences between residents living in the central business district, along the scenic Brisbane River, in sprawling suburbs, and in outlying rural communities. The first study contrasting leukocytes from late-term pregnant women and their newborn children provides strong evidence for transmission of two classes of gene expression profile that are orthogonal to the differentiation of maternal and cord blood samples. The second study evaluating leukocyte gene expression profiles of 100 Red Cross blood donors confirmed that two less prominent axes of variation in the mother-newborn comparison are more generally seen in gene expression profiles of Red Cross blood donors, one of which is the similar to the major component of variation distinguishing rural and urban residents in southern Morocco [19], while a hint of geographic structure was also evident in the sample. These data confirm that peripheral blood gene expression does vary somewhat discretely even in urban populations, in part reflecting metabolic phenotypes of the adult population, with implications for early influences on susceptibility to chronic disease.

## Results

### Mother-Newborn Study

The leukocyte gene expression profiling dataset for the mother-newborn study consisted of 56 individuals, with 19 mothers sampled in the last month of pregnancy and 37 cord blood samples from newborn babies. Nine of the mothers were overweight, most of whom had BMI values over 30 (before pregnancy) and hence were classified as obese. From the 37 children, 11 were born to obese mothers, 8 to gestational diabetic mothers (with a wide range of body mass indices), and 18 to normal-weight mothers. There were 16 mother-newborn pairs (that is, 32 of the 56 individuals) in the dataset.

**Figure 1:**
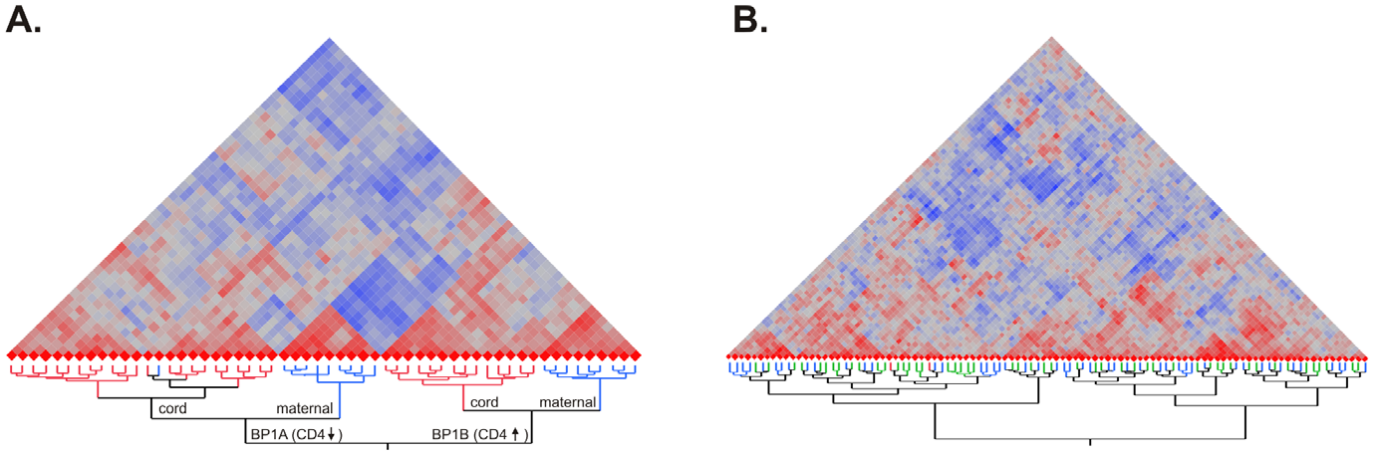
Correlation structure of leukocyte profiles. Heat maps show overall strength of pairwise correlation between pairs of profiles (red, high positive, blue, high negative). (A) In the mother-newborn study, clusters of samples obtained from cord blood (red lines) and maternal (blue lines) are clearly evident within the two larger clusters, BP1A and BP1B. (B) In the Red Cross study, two large clusters are observed that are unrelated to gender (blue, male; green, female) with further structure visible within both major clusters.

After normalization and adjustment for RNA integrity, we examined the patterns of relatedness among individuals, first by clustering the overall profiles. As shown in Figure 1A, there are two clearly visible levels of clustering. The highest level distinguishes two types of profile, labeled BP1A and BP1B, and analysis of variance indicates that 8,428 transcripts differ between these two groups of individuals at a false discovery rate of 1% (and 3,983 transcripts at p<10^−5^). Within each of BP1A and BP1B, there is also clear separation of the profiles of mothers and newborns; ANOVA identifies 7,370 transcripts of this class at the 1% FDR (3,475 at p<10^−5^). There was no evidence for any influence of generation on the BP1 profile as fewer probes than expected by chance show an interaction effect at any level of significance. This implies that whatever factors drive the differentiation of BP1A and BP1B are independent of those that distinguish mothers and newborns, and influence non-overlapping suites of genes.

The two BP1 profiles do not correlate with any epidemiological covariates that we have considered: they are not representative of maternal weight or age, and only a handful of the mothers had health conditions that might have modified their expression profiles. Astonishingly, though, we observed perfect transmission of the two profiles from mother to newborn: all 7 BP1A mothers with newborns in the dataset had BP1A newborns, and all 9 BP1B mothers had BP1B newborns. This result indicates a strong tendency to inheritance of overall leukocyte gene expression profiles, but our data does not establish whether the mechanism is genetic or environmental. Nor is it clear whether the profiles are stably retained through the lifetime of the individuals.

A more formal quantitative genetic test of inheritance was performed by indexing each of the 16 mother-newborn pairs with a parameter indicating their familial relationship. An ANOVA on this reduced dataset of 32 individuals showed that the pair index explains half of the variance in the first principal component of transcript abundance, suggesting very high heritability. However, this approach almost certainly over-estimates the genetic contribution since environmental factors likely contribute to the separation of BP1A and BP1B, thereby contributing to the observed similarity between parent and child. If it is instead assumed that this separation is entirely due to environment, then the minimal genetic contribution can be estimated as the proportion of variance explained by the pair index when both generation, and BP1A or BP1B status, are included in the model. When this is done, BP1 status explains 45% of the variance in gene expression, while the mother-child pair index explains just 3%, indicating that shared inheritance only explains a small proportion of the variance within the two BP1 profiles. In other words, within BP1A or BP1B, mothers and newborns only share slightly more similar profiles than randomly chosen pairs of individuals, but the clear differentiation of these classes nevertheless represents very strong transmission of leukocyte expression from mother to newborn. It is not possible from these analyses to infer the true contribution of genotype to the separation between the profiles.

**Figure 2:**
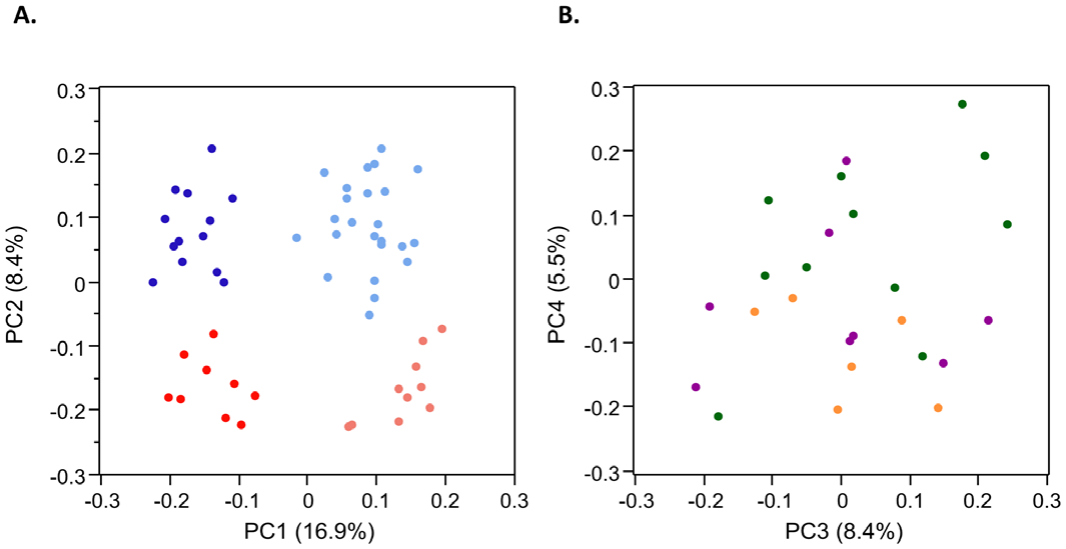
Principal Components (PC) of expression variation in the Mother-Newborn study. (A) PC2_MN_ cleanly separates maternal (red) and cord blood (blue) samples, but an even greater proportion of the variation and separation of profiles is observed for PC1_MN_, which defines the BP1A (dark) and BP1B (light) types discussed in the text. (B) PC3_MN_ is not associated with any measured phenotypic variable, but PC4_MN_ largely distinguishes cord blood of obese children (green) and gestational diabetic children (purple), while maternal blood (orange) also has negative values of PC4_MN_.

We next performed principal component (PC) analysis on the full dataset, and observed that the first five PC explain 50% of the total variance in gene expression. PC1_MN_ (16.9%) corresponds to the BP1A versus BP1B difference, while PC2_MN_ (15.9%) corresponds to the Mother-Newborn distinction (Figure 2A). The next three PC explain 8.4%, 5.5% and 4.0% of the variance respectively. The numbers of probes significantly associated with each PC are reported in Table 1. None of the PC are significantly associated with maternal body weight, although a suggestion that PC4_MN_ reflects metabolic status is indicated in Figure 2B, which shows that newborn babies of obese mothers typically have positive values of PC4_MN_ (green spots), while obese mothers (orange spots) all have negative values of PC4_MN_. This contrast is significant (p = 0.002, 2-tailed *Fisher*'*s exact* test), and implies a switch in this profile between maternal and cord blood. Intriguingly, babies of gestational diabetic mothers (purple spots) also tend to have negative values of PC4 (p = 0.02, 2-tailed *Fisher*'*s exact* test). Normal mothers and newborns are not indicated on Figure 2B as they cover the full range of values for PC3_MN_ and PC4_MN_. PC5_MN_ is specific for some of the newborn babies, one third of whom have lower values than any of the mothers.

**Table 1:** Numbers of genes significantly differentially expressed in each profile.

Significance Level		Mother-Newborn Study				Red Cross Study		
	BP1	M-N	PC3_MN_	PC4_MN_	PC5_MN_	PC1_RC_	PC2_RC_	PC3_RC_
p<10^−5^	3983	3475	2906	1253	363	4769	3100	1866
q<0.01 (FDR 1%)	8428	7370	6613	5016	3264	9096	7149	5789
p<0.05	10107	9059	8049	6609	4915	10095	8345	7065

BP1 and M-N are categorical contrasts; all other are regression of expression on the PC.

### Red Cross Study

In order to determine whether the expression profiles differentiating pregnant women and their babies are also present in a general population of adults in Brisbane, we generated similar profiles for a sample of 100 Red Cross blood donors. Hierarchical clustering (Figure 1B) and principal component analyses once again identified major axes of variation, but in this case the first three axes, PC1_RC_, PC2_RC_, and PC3_RC_ explain just 10.9%, 8.3% and 5.6% of the variance respectively. We obtained BMI measures for all donors, and assayed for presence or absence of cytomegalovirus and Epstein-Barr virus infection, but none of these factors were correlated with the PC axes of variation.

**Figure 3:**
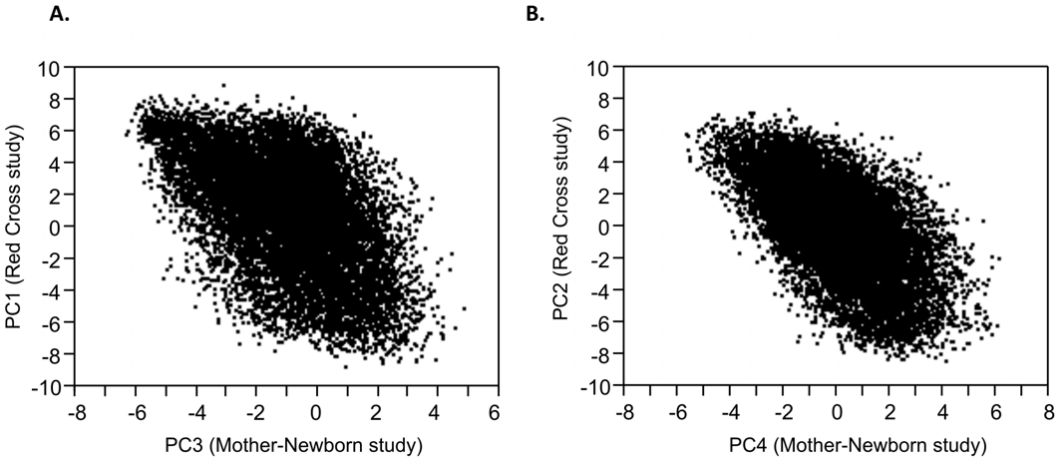
Similarity of major axes of variation in the two studies. (A) Scatterplot of PC loadings for PC3_MN_ and PC1_RC_: slope  = 0.69, *R^2^* = 0.35, although a large number of uncorrelated probes are also observed (-2<PC3_MN_ <2) (B) Scatterplot of fold PC loadings for PC4_MN_ and PC2_RC_: slope  = 0.72, *R^2^* = 0.37.

Although the two samples were collected at different times, and processed independently, there is highly significant overlap in the patterns of differential gene expression associated with the common axes of variation. This is seen in evaluation of the correlation between the slopes of the multiple regression co-efficient for each PC (Table 2) and Figure 3 for PC3_MN_ and PC1_RC_, and for PC4_MN_ and PC2_RC_. Note that PC2_MN_ corresponds to the mother-newborn distinction that is not present in the Red Cross study. Surprisingly, PC1_MN_ was not highly correlated with any of the axes in the Red Cross study, although many of the most divergent genes between the profiles also contribute to differentiation in the other components. PC5_MN_ and PC3_RC_ do not correspond to one another. There are 1239 genes significantly correlated with both PC3_MN_ and PC1_RC_ at p<10^−5^, the vast majority of which are up-regulated 2-fold or more in half the individuals. For the axis corresponding to PC4_MN_ and PC2_RC_, there are 656 transcript probes in common at p<10^−5^, each of which shows the same direction of differential expression in the two studies (291 down and 365 up).

**Table 2:** Similarities between PC axes in the two studies.

	PC1_MN_	PC2_MN_	PC3_MN_	PC4_MN_	PC5_MN_	PC1_RC_	PC2_RC_	PC3_RC_
PC1_MN_		**0**	0	0	0	0.08	0.23	0.02
PC2_MN_	**0**		0	0.19	0.18	0	0.32	0
PC3_MN_	−0.03	−0.08		0	0	0.29	0.05	0.06
PC4_MN_	0	−1	0.03		0.04	0	0.39	0.03
PC5_MN_	0.01	−0.74	−0.04	0.15		0.02	0.16	0.08
PC1_RC_	−0.28	0.03	−0.97	0.11	0.33		0	0
PC2_RC_	−0.56	2.07	0.35	−1.01	−0.84	0		0
PC3_RC_	−0.11	0.03	0.29	−0.2	0.45	−0.01	0.03	

Adjusted R-squared above diagonal; Slope of regression below.

**Figure 4:**
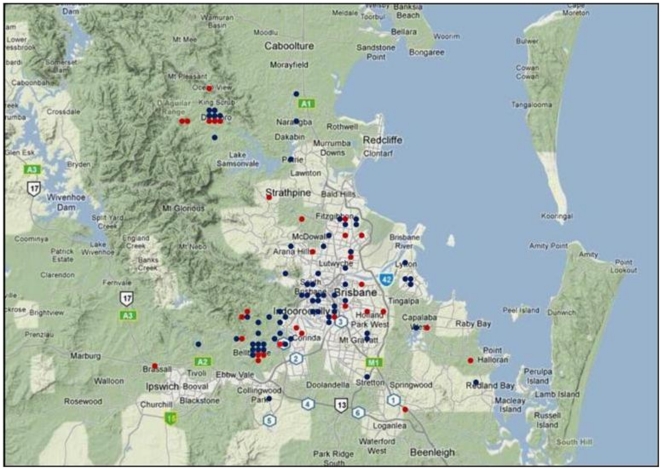
Distribution of PC3_RC_-associated expression module across the city of Brisbane. Blue and Red dots correspond to individuals with high and low values of PC3_RC_ in the Red Cross study, superimposed on a GoogleMap of the city. There are 199 genes down-regulated and 209 upregulated at p<10^-5^ between two major profiles. Note that the blue profile is distributed throughout the city, while the red one is excluded from the central business district (near South Brisbane on the map) and along the riverfront extending southwest to Indooroopilly. Green shading indicates more rural regions surrounding the city, whereas yellow indicates suburban and high density areas. Blue is Moreton Bay, with the Pacific Ocean to the east (right).

BP1A and BP1B are distributed randomly across the geography of Brisbane, but the profiles associated with PC3_RC_ show a non-random distribution as shown in Figure 4. One of the profiles, indicated by the red dots, is excluded from the central portion of the city along the Brisbane River (*p* = 0.005, 2-tailed *Fisher*'*s exact* test), while the other is found throughout the city and surrounding regions. This tendency is noteworthy given a socio-economic differentiation between the city and riverfront (which tend to be more affluent) and outlying suburbia. Obesity levels are not significantly different between the two parts of the city in the sample, and do not associate with the two profiles (*p* = 0.88, *t*-test). Moreover, the profile observed to switch between the obese mothers and their babies in the first study, corresponding to PC2_RC_, is not geographically structured in this study. All principal component loadings and significance values are listed in Table S1.

### Gene Content of the Profiles

Gene Ontology analysis summarized in Table 3 generally identified broad classifications of gene function as responsible for the major axes of variation, with the most significant terms involving intracellular protein trafficking, protein modification, and organellar biogenesis. A sharp contrast was observed in the differentiation between maternal gene expression and cord blood leukocytes, which largely involves a variety of immune pathways, cytokine signaling, and antiviral defense, as noted by Merkerova et al. [12] and Jiang et al. [13]–[14]. We observed higher expression (p<10*^−5^*) in maternal than cord blood of transcripts related to Interleukin (17 genes vs 5 genes) and Interferon (15 vs 3) signaling, as well as of Toll-like receptors (7 vs 0) and the TRIM family of protein-degradation enzymes (13 vs 4). Similar numbers of CD and C-lectin transcripts were differentially expressed in mothers and newborns, but cord blood shows higher expression of MRP multidrug resistance genes (21 vs 1), ribosomal proteins (16 vs 0 RPS and RPL genes), WD-repeat genes (11 vs 2), the MCM replication initiator complex (7 vs 0), EIF translation initiator proteins (11 vs 3) and NDUF complex NADH: ubiquinone oxidoreductases (13 vs 1). These results are consistent with the notion that the leukocyte population in newborns is poised for expansion, while adult leukocytes are more prepared to mount an immune response. Differential expression values are also provided in Table S1.

**Table 3:** Enriched gene sets.

Category	Term	Count	P Value	Fold Enrich	Benjamini
Mother-Newborn PC1 (BP1A versus BP1B)					
GOTERM_CC_ALL	GO:0005622∼intracellular	510	1.09E-28	1.3	4.75E-26
GOTERM_CC_ALL	GO:0044422∼organelle part	209	9.82E-14	1.6	1.07E-11
GOTERM_BP_ALL	GO:0044238∼primary metabolic proc	376	1.36E-10	1.2	1.78E-07
GOTERM_BP_ALL	GO:0015031∼protein transport	64	2.19E-11	2.5	5.75E-08
GOTERM_BP_ALL	GO:0016043∼cell component biogen	159	2.99E-10	1.6	3.14E-07
GOTERM_CC_ALL	GO:0043233∼organelle lumen	66	7.91E-07	1.9	4.58E-05
GOTERM_CC_ALL	GO:0005794∼Golgi apparatus	51	8.66E-07	2.1	4.42E-05
GOTERM_BP_ALL	GO:0006396∼RNA processing	43	3.58E-08	2.6	1.34E-05
GOTERM_CC_ALL	GO:0012505∼endomembrane system	67	9.87E-07	1.9	4.76E-05
GOTERM_BP_ALL	GO:0010467∼gene expression	182	3.29E-06	1.4	7.85E-04
GOTERM_BP_ALL	GO:0043687∼post-translational mod	87	2.38E-05	1.6	0.004
Mother-Newborn PC2					
GOTERM_BP_ALL	GO:0002376∼immune system process	70	6.92E-09	2.1	3.64E-05
GOTERM_CC_ALL	GO:0005622∼intracellular	350	1.38E-06	1.2	3.99E-04
SP_PIR_KEYWORDS	antiviral defense	9	1.29E-05	8	0.0034
SP_PIR_KEYWORDS	erythrocyte	6	7.11E-05	12.9	0.0108
Mother-Newborn PC3 and Red Cross PC1					
GOTERM_CC_ALL	GO:0033279∼ribosomal subunit	45	4.63E-37	13.2	1.34E-34
GOTERM_CC_ALL	GO:0044444∼cytoplasmic part	167	1.23E-18	1.9	1.07E-16
KEGG_PATHWAY	hsa00190:Oxidative phosphorylation	22	6.95E-08	4	6.99E-06
GOTERM_BP_ALL	GO:0016043∼ cell component biogen	108	3.13E-08	1.7	3.29E-05
GOTERM_CC_ALL	GO:0005773∼vacuole	18	2.80E-05	3.4	9.36E-04
GOTERM_BP_ALL	GO:0016192∼vesicle-med transport	30	1.37E-05	2.5	0.0048
GOTERM_BP_ALL	GO:0030029∼actin filament process	18	3.05E-05	3.3	0.0094
GOTERM_BP_ALL	GO:0006915∼apoptosis	37	1.27E-04	2	0.0263
SP_PIR_KEYWORDS	myristylation	8	4.20E-05	8.3	0.005
Mother-Newborn PC4 and Red Cross PC2					
KEGG_PATHWAY	hsa04660:T cell receptor signaling	14	1.22E-06	5.4	2.46E-04
GOTERM_CC_ALL	GO:0042101∼T cell receptor complex	5	7.53E-05	20.3	0.0322
GOTERM_CC_ALL	GO:0001772∼immunological synapse	6	4.22E-05	14.6	0.036
**Red Cross PC3**					
GOTERM_CC_ALL	GO:0005622∼intracellular	283	1.25E-16	1.3	9.64E-14
GOTERM_CC_ALL	GO:0044428∼nuclear part	55	5.35E-10	2.5	7.74E-08
GOTERM_MF_ALL	GO:0003723∼RNA binding	45	2.08E-11	3.2	5.98E-08
GOTERM_BP_ALL	GO:0044267∼cellular protein metab	112	1.98E-08	1.6	3.46E-05
GOTERM_BP_ALL	GO:0048523∼neg. regulation cell proc	42	1.13E-04	1.9	0.0226
GOTERM_BP_ALL	GO:0006915∼apoptosis	33	7.97E-05	2.1	0.0188
GOTERM_BP_ALL	GO:0015031∼protein transport	32	2.09E-05	2.3	0.0064
GOTERM_BP_ALL	GO:0007242∼intracell signaling cascade	54	1.79E-05	1.8	0.0063
KEGG_PATHWAY	hsa05220:Chronic myeloid leukemia	10	5.54E-05	5.6	0.0111

Notably too, the major axis of variation in Brisbane adults (PC1_RC_ and PC3_MN_) is highly enriched for oxidative phosphorylation and ribosomal protein genes. This axis is reminiscent of the major axis of variation differentiating rural and urban residents in southern Morocco [19], although the involvement of *SNORD* family RNAs seen there was not observed in Brisbane. The second axis (PC2_RC_), corresponding to the module that apparently switches between obese mothers and their children (PC4_MN_) is exceptional in showing no enrichment for general cellular processes, but rather identifies differential expression of the T cell receptor complex and signaling components, as well as of *CD8* transcripts.

No formal statistical relationship between body mass index, body weight, or gestational diabetes and the expression of individual genes was observed, but targeted analysis of specific pathways hints that there is some connection between metabolic status and leukocyte gene expression. Among the most highly represented KEGG pathways in several of the signatures are insulin signaling, and various aspects of central metabolism. Monocytes and macrophages are major targets and producers of IGF-1, which is implicated in cardiovascular, metabolic, and lung disease [21], but it is the *IGF2R* receptor transcript that is most strongly associated with the principal components of expression in both studies. Functional variation in the IGF pathway seems a promising target for focused evaluation of the personal health implications of peripheral biomarkers and their response to environmental agents such as smoking and diet. Indeed, this theme is reinforced with the differential expression of multiple genes in KEGG pathways involved in cell-environment (focal adhesion/actin cytoskeleton, MAPK and neuronal disease pathways) and cell-proliferation processes (Cancer, AML and MAPK pathways).

**Figure 5:**
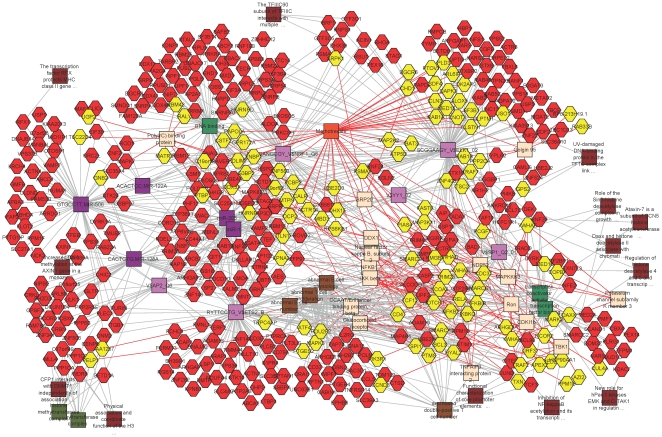
Cluster analysis of BP1 using the ToppGene Suite. The top 1,000 transcripts contributing to BP1 (PC1_MN_) were scanned for significant enrichment of transcription factor and microRNA binding sites and targets of the major factors were clustered using Cytoscape. Red hexagons show predicted targets of the transcription factors in light purple squares (ELK1, SP1, ETS2, NRF1, AP2 and YY1) as well as the micro RNAs in dark purple squares (miR-206, miR-1, miR-506, miR-128A, and miR-122A). Dark brown and green boxes highlight chromatin modification factors including DNA and histone methylases and acetyltransferases. Methotrexate-responsive genes are indicated in yellow.

We also used the ToppGene suite [22] to search for over-representation of predicted transcription factor binding sites and/or microRNA binding sites in the genes that account for each principal component of variation. As a measure of robustness, we looked for enrichment both in the top 1000 most significant genes for each PC, and for all genes with p<10^−5^ (Table S4). Three transcription factors emerged as significant for BP1 in the mother-newborn dataset: ELK1, NRF1, and ETS2 with 113, 82 and 84 targets respectively, while another 5 transcription factors were significant only in the top 1,000 gene set. Two micro-RNAs, miR-206 and miR-1 each had 50 differentially expressed targets among the approximately 400 known transcripts with predicted 3′ UTR binding sites. Figure 5 shows a cluster analysis of these genes, and also indicates (in yellow) a suite of transcripts that are known to be responsive to methotrexate, which is one of two drugs whose targets are significantly over-represented in the top 1,000 most significant genes for BP1 (p<0.00002), the other being Vitamin E. Various genes highlighted in brown encode proteins involved in chromatin modification, including the methyltransferase DNMT1.

ToppGene suggested enrichment for transcription factor target sets in PC3_MN_ and for PC4_MN_, both also implicated in regulation of BP1 (PC1_MN_), namely ELK1 and ETS2 respectively. No miRNA were consistently associated with these PC, or with PC2_MN_, which distinguishes maternal and cord blood. However, each of PC3_MN_ and PC4_MN_ are enriched for suspected targets of over 50 compounds, most of which are unique for each PC, including such toxins as paraquat, arsenic and ozone; drugs such as Thalidomide, Hydralazine, and Valproic acid; and herbicides including Clofibric acid, Isoproturon, and Atrazine. Furthermore, the association of PC4_MN_ with metabolic regulation is supported by the significant enrichment for altered expression of 19 of the 68 genes annotated in the Reactome “metabolism of sugars” pathway, including such enzymes as G6PD, GAPDH, and PGK1. Many other potential targets of a half dozen transcription factors and 35 microRNAs were associated with the major PC of the Red Cross blood donor dataset.

## Discussion

### The Structure of Leukocyte Gene Expression Variation in Brisbane

We document a considerable degree of population structure in a diverse adult population of residents of a large city in a developed country, extending our previous finding that over a third of the leukocyte transcriptome differs between urban and rural residents of Morocco [18]. Four aspects of the analysis are particularly noteworthy: (1) the first principal component in mothers and newborns is unique to these individuals, but overlays two additional components of variation that were replicated in our study of adults blood donors; (ii) the unique profile reflecting *CD4* transcript abundance appears to be faithfully transmitted from pregnant mothers to their newborn children; (iii) a profile involving several hundred genes may differentiate newborn babies of obese and gestational diabetic mothers; and (iv) a minor component of variation also shows a hint of demographic differentiation between the city itself and surrounding suburbs.

Gene expression differences in a complex cell population such as peripheral blood leukocytes are likely to reflect differences both in the relative abundance of major cell types (such as lymphocytes, neutrophils, and macrophages), and differential expression within cells. Platelets and erythrocytes were removed by filtration during sampling, but the leukocyte samples are nevertheless diverse, so we also estimated the number of cells expressing various CD (cellular differentiation) antigens by flow cytometry for half of the adult blood donor sample. CD8 transcript and cell counts were highly correlated (n = 47; p<0.0001 for each of 6 different *CD8A* and *CD8B* probes), but this was not true of CD4 (p = 0.08 for the single probe) and hence the ratio of these cell types is not directly reflected in the ratio of transcript levels. This probably reflects the diversity of CD4+ cells, as well as the fact that cell counts are independent of marker intensity. Unfortunately, we did not have FACS data or complete blood counts for the mother-newborn study and cannot conclude whether the expression divergence reflects altered fractions of contributing cell types.

**Figure 6:**
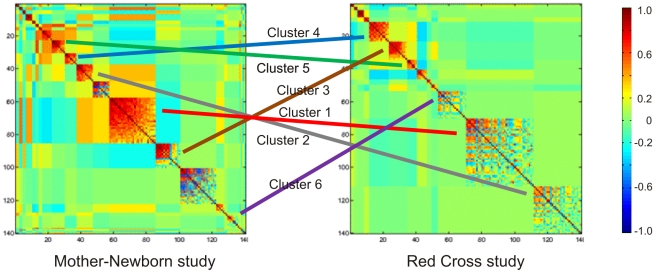
Consistent clustering of cell surface marker transcripts. The MMC optimal clustering procedure was used to identify groups of co-regulated CD, C-type lectin, and Toll-like receptor transcripts in the two studies. Lines link the indicated clusters consisting of genes listed in Table 4. Clusters 1B and 2B correspond to the more tightly co-expressed genes in the larger clusters seen in the figure. Color key indicates strength and sign of correlations within and between clusters.

Independent evidence that differences in cellular abundance contribute to expression variation can be seen by evaluating the correlation structure of transcript abundance across all of the 103 CD, 26 C-type lectin, and 11 Toll-like receptor probes that showed expression above background. Modulated modularity clustering [23] of these profiles in both datasets revealed eight strongly co-regulated groups of transcripts (Figure 6). These also cluster for the most-part in our Moroccan dataset (not shown), and are recapitulated using classical hierarchical clustering with the difference that positively and negatively co-varying transcripts cluster together in the MMC analysis. The largest cluster (Cluster 1) includes most of the TLR, CLEC4E, and ten CD genes including CD4; CD8 clusters with CD2 and CD3D,G; and the other major CD clusters are listed in Table 4. Such clustering can be readily explained assuming that the respective transcripts are indicative of common cell surface protein expression on particular differentiated cell types. The analysis also implies that genes in Clusters 4, 5 and 6 are expressed not just independently of, but to some extent to the exclusion of those in Cluster 1. Co-regulation of the C-type lectin and Toll-like receptors in cluster 1B (the most strongly correlated transcripts represented by the bright red coloring in Figure 6) is likely to have functional implications with respect to the ability to respond to fungal and bacterial pathogens [24]–[25], particularly if the recruitment of particular T-cell classes is affected by constraints on gene expression that are seen in the observed correlation structure.

**Table 4:** Co-expressed clusters of cell surface markers.

Cluster 1A	CD4	CD3D	CD3E	CD37	CD74	CD99	CD164	CD320	
Cluster 1B	CD36	CD46	CD55	CD58	CD84	CD86	CD93	CD302	
	CLEC4A	CLEC4D	CLEC4E	TLR1	TLR2	TLR4	TLR5	TLR6	TLR8
Cluster 2A	CD14	CD48	CD53	CD68	CD97	CD99	CD300A	CD300LB	
Cluster 2B	CD2AP	CD2BP2	CD47	CD63	CD69				
Cluster 3	CD19	CD22	CD40	CD72	CD79A	CD79B	CD83		
Cluster 4	CD8A	CD8B	CD2	CD3D	CD3G				
Cluster 5	CD7	CD96	CD160	CD247					
Cluster 6	CD1A	CD1C	CD200R1	CLEC10A					

All clusters observed reported in this table were observed in both datasets.

A and B refer to subclusters within 1 and 2, and generally include positively or negatively correlated sets of transcripts.

The transmission of the BP1A and BP1B profiles from mother to newborn could conceivably reflect genetic or environmental regulation of the abundance of cell types as well, though if the effect were genetic it would be unlikely to be so clearly maternally transmitted. CD4 to CD8 cell surface ratios have been shown to be heritable in a large cohort of Brisbane adolescent twins [26]–[27], and to be associated with Type 1 Diabetes as well as control of HIV infection. In the present study the ratio of transcript abundance of these two genes is extremely highly associated with BP1A or BP1B profile (*F*_1,54_ = 58.8, *p* = 3×10^−10^), but this is mostly due to variation in CD4 transcripts, while CD8 expression varies along the PC4_MN_ and PC2_RC_ axes. Unexpectedly, the BP1 profile defined by PC1_MN_ is not observed in the more general adult cohort, raising the possibility that it is unique to pregnant women and their children. The possibility that it is a technical artifact of the analysis must be considered, but it also dominates the profiles of only the individuals with the highest quality RNA (see methods), it is hard to conceive that an artifact would stably transmit across the generations, and would so clearly correlate with a measure of differentiation of major T cell classes. Each individual's recent history of immune exposure could conceivably shift the expression profiles between modes, but since the fetal immune system is not thought to be responsive to pathogens, our data suggests instead that some aspect of the maternal environment that does cross the placenta barrier (such as cytokine profiles, perhaps) has a major impact on leukocyte gene expression in both the mother and newborn.

The major axis of variation documented here in the cross-sectional adult study (PC1_RC_) appears to correspond to the major axis we observed differentiating remote Berber villagers from urban Moroccans using basically the same leukocyte sampling strategy [19]. In Brisbane, the separation is not obviously geographically structured, indicating that the environmental factor driving the qualitative differentiation is embedded in lifestyle differences even among residents of a large modern city. Enrichment of genes involved in oxidative phosphorylation and ribosomal biogenesis is suggestive of cell growth and division, consistent with the fact that although circulating monocytes and macrophages are post-mitotic, peripheral T-cells will divide in response to antigenic stimulation and neonate T-cells are highly proliferative [28]. Another possible source of differentiation along this axis is a direct impact of antioxidants and/or renal stress. It should be emphasized as well that, although present in mothers, this particular profile was not transmitted from mother to newborn as it randomly assorts among the mother-baby pairs.

We did not observe the correlation of gene expression with obesity reported in peripheral blood monocytes in a larger Icelandic cohort [16] In fact, we specifically tested for association between individual transcripts and either BMI (in both studies) or metabolic syndrome indicators (obesity, gestational diabetes, normal weight in the mother-newborn study), but there was not an excess of significant associations relative to random expectations. It thus seems likely that the major environmental influences on gene expression profiles are to some extent population-specific. Broader sampling across a diversity of lifestyles will be required to detect commonalities, and to define baseline levels of variation with which to detect abnormality in specific cohorts.

### The Maternal Origins of Disease

The original ‘thrifty phenotype’ hypothesis [2] was devised to explain the observation of elevated rates of obesity and early onset diabetes in children born to undernourished mothers. It suggested that metabolic reprogramming occurs to prepare children for a hostile nutritional world, and has given rise to a school of thought that mismatches between maternal and external environments commonly contribute to chronic disease [29]–[30]. More generally still, it is now recognized that maternal health, independent of genetics, is a major contributor to child development and the onset of disease [1]. If this is true, then we would expect to see an influence of maternal metabolic status already at birth, for example in the profiles of expression in cord blood.

Our data provide some support for this idea. Most pointedly, there is unambiguous transmission of the major maternal profile, BP1A or BP1B, from mother to newborn. The separation of the two profiles is actually stronger in the newborns than the mothers and was not detected in the general male and female adult population (although the most divergently expressed genes are enriched in the second component of variation in the second cohort of adult blood donors). At this point, we do not know to what extent the profile is stably maintained through the life of an individual, and longitudinal studies will be required to monitor this and ascertain whether there is any prospective impact on the likelihood of development of chronic disease. Importantly for a strict version of the thrifty phenotype hypothesis, there is no correlation between BP1 status and obesity or gestational diabetes, so the profile is not a discriminative biomarker for these two metabolic diseases.

It may however be a contributor, since insulin signaling and sugar metabolism are among the key regulated pathways and are also affected by the minor axes of variation, one of which (PC4_MN_) appears to distinguish babies born to obese and gestational diabetic mothers (Figure 2B), albeit in a small sample to date. If replicated, this observation would provide a compelling argument for reprogramming of expression states in leukocytes, particularly given the switch in status between obese mothers and their children. One interpretation of our data is that obese mothers have adapted to their metabolic status by shifting their own expression profile to a more normal state that is also seen in children born to gestational diabetic mothers who are unable to transmit the reprogramming signal to their newborns. Normal weight mothers themselves do not show any tendency to have a specific profile, and nor do their children (data not shown). Children born to mothers with the lowest BMI in some cases had profiles similar to those of the children born to obese mothers, consistent with the observation that both low and high weight mothers are at risk of having children with early onset metabolic syndromes. Importantly, the maternal risks are statistical tendencies, so we do not necessarily expect strong correlation between expression states in newborns and the development of metabolic disease, and it may take samples of several thousand mother-baby pairs to establish a relationship.

It would be premature to extrapolate from this pilot study to conclude that maternal health impacts child health already through reprogramming of gene expression in fetal tissues. However, our analyses do provide the first genomic evidence that this may be the case, and calls for replication in larger and more diverse settings. The suggestion that differential expression of methotrexate-responsive genes is common in pregnant mothers is also relevant from to child health, since methotrexate inhibits tetrahydrofolate production, and low levels of this vitamin B9 derivative are known to cause spina bifida; most expectant mothers (including all in this study) are encouraged to take folate-supplements to prevent this condition. It is also interesting to contemplate the implications of the different leukocyte gene expression profiles for immune function in the first few months and years of life. Categorical differences in immune profiles may contribute to susceptibility to the development of inflammatory, autoimmune, and aberrant infectious disease responses, and may impact on the use of cord blood cells in transplantation.

## Materials and Methods

### Blood Collection and RNA extraction

This study was approved by Institutional Review Boards of the University of Queensland and the Wesley Research Institute, and was conducted with permission of the Red Cross Society of Australia. Written informed consent was obtained from all participants and informed assent was provided by parents of newborn children. Blood was collected by venipuncture (or extraction from the umbilical cord immediately after birth). Within 2 minutes of sampling, between 5 and 10 ml of blood were washed through a LeukoLock size-fractionation filtration system (Ambion/Applied Biosystems, Austin TX) that traps leukocytes while allowing most platelets, erythrocytes, and serum to pass through. Following manufacturer's recommendations, the filters were immediately infiltrated with RNAlater solution and stored frozen at −20°C until preparation of RNA.

RNA samples for the mother-newborn study were collected following informed consent from pregnant mothers enrolled as patients in the medical practice of Graham Tronc, ObGyn, at Brisbane Private Hospital. All pregnant mothers in this study were encouraged to take a vitamin supplement rich in folic acid, either Blackmore's Gold Pregnancy, Elevit, or Fefol, but no data on intake is available. Maternal samples were collected between the 30th and 36th week of pregnancy during regular visits and were processed through the LeukoLocks by staff of Gribbles Pathology Associates. Umbilical cord blood samples were processed at birth by GG or EM, with the majority of deliveries by caesarian section arranged in advance between the hours of 8am and 11am. All samples for this study were collected between August 2008 and February 2009. In order to avoid batch effects, we did not extract RNA until immediately prior to microarray hybridizations after all samples had been collected. At this time, we discovered that a large number of the samples had been affected by significant RNA degradation with the result that only 56 of the 90 originally collected samples yielded RNA of minimal acceptable quality for hybridizations. The cause of the degradation is unknown, but we suspect a change in the pH of the PBS or RNAlater solutions used for washes over several months and/or variation in the sealing of the filters before storage by multiple individuals engaged in the processing. As a consequence, only 16 mother-newborn pairs were included in the study, with the remaining 24 samples from only the mother or baby.

Blood samples for the cross-sectional study were collected with permission of the Red Cross Society of Australia and under informed consent from 100 participants (50 men and 50 women) between the ages of 18 and 68 (mean 44). BMI ranged from 17.7 to 51.3 with a mean of 26.6. Collection was carried out by EM at mobile Red Cross vans at 10 locations (Bellbowrie, Capalaba, Dayboro, Eagle Farm, Kenmore, Mount Ommaney, South Brisbane, St Lucia, Virginia, and Woolloongabba) distributed across the city, most with multiple sampling days and 5–10 samples per day, between March and June, 2009. Each of these samples was also tested for the presence of antibodies against EBV and CMV using a rapid ImmunoDOT Mono-G test-strip assay (GenBio, San Diego CA). Individuals also provided a limited amount of information by written survey regarding their location of residence, age, gender, weight and height, and recent pharmaceutical usage. Sample features are listed in Table S2.

Following extraction of total RNA from the LeukoLock filters according to manufacturer recommended protocols, the RNA concentration was estimated with a NanoDrop 1000 spectrophotomer and quality was assessed with an Agilent Bioanalyzer. RIN (RNA Integrity Number) scores for the samples included in the mother-newborn study ranged from 3.2 to 9.3 (median 6.75; 23 samples greater than 7.0). RIN scores for the samples included in the Red Cross study ranged from 4.9 to 9.5 (median 8.75; 66 samples greater than 8.0).

### Flow Cytometry

In order to estimate the relative abundance of cells, flow cytometry was performed on a BD LSR-II at the Queensland Brain Institute. EDTA treated whole blood was incubated in FACS Lysing Solution (BD Biosciences, San Jose, CA) and stained with diagnostic monoclonal antibodies (BD Biosciences) against seven different cell-surface Cellular Differentiation antigens: CD3, CD4, CD8, CD16, CD19, CD45, and CD123. Each batch of samples was kept at room temperature, and processed the day immediately following blood collection, such that no samples were frozen or stored for longer than 24 h at room temperature. An unstained control was also tested for each sample, as well as positive controls for each of the seven antibodies from one of the samples in each batch.

Cell counts were analyzed using Weasel 2.6.1 software developed by the Walter and Eliza Hall Institute (Melbourne, Australia) downloaded from http://en.bio-soft.net/other/WEASEL.html. Contour plots for pair-wise comparisons of channel intensities were drawn, and 91.67% densities selected manually. Samples were included where the sum of CD4+ and CD8+ was within 85% of the CD3+ count, and where the sum of CD3+, CD16+ and CD19+ cells was greater than 80%. CD4 averaged 14.3% with a standard deviation of 4.0%, and CD8 averaged 6.7% with a standard deviation of 3.7%. Similar results were obtained using the automated gating options with BD's FACSDiva software.

### Gene Expression Profiling

Each RNA sample was reverse transcribed and labeled with Cy3 dye according to standard Illumina protocols (using 500 ng of RNA and 14 h incubation for the IVT reaction), and hybridized to an Illumina HT-12 bead array with over 48,000 probes, each represented by between 20 and 80 individual beads. Two blocks of hybridizations were performed, one for each study, a couple of months apart. Within each study, samples were randomized across the arrays with respect to maternal or newborn origin of the sample, or with respect to collection site and location of residence. Arrays were scanned on an Illumina BeadArray Scanner and data was extracted with Genome Studio Software. Standard array quality measures indicated high quality hybridization and all samples were taken forward for further analysis. The mother-newborn study included one technical replicate (both samples cluster adjacent to one another in all subsequent analyses), and the Red Cross study included six technical replicates (four of which cluster adjacent to one another, one very close, while fifth was disparate but in the same broad profile group).

### Statistical Analyses

Raw probe summary data was exported into Microsoft Excel and transformed on the log base 2 scale. Each study was analyzed separately. In order to reduce the dataset to include only genes that are expressed above background, we first computed the average expression level for each probe across all of the samples, and plotted these averages in rank order. The inflection point of this curve suggested a conservative cutoff including 15,000 probes for each study, of which 13,715 (91.4%) representing 10,987 different genes were included in both analyses. The log_2_ values for each of the 15,000 probes were then imported into JMP Genomics 4.0 (SAS Institute, Cary NC) for all subsequent analyses. These values are available as Table S3, and the raw array measures are available at the Gene Expression Omnibus (GEO) repository as series GSE21345 with sub-series GSE21311 and GSE21342 for the Red Cross and Mother-Newborn studies respectively.

JMP Genomics provides a versatile analysis environment with workflows for performing quality control and data normalization, supervised and unsupervised clustering, and analysis of variance of gene expression profiles. Initial exploratory analyses indicated that the raw data is influenced by a variety of technical artifacts, principally RNA quality (samples below RIN 6.5 cluster quite distinctly from those above RIN 6.5), and in the Red Cross study an array effect that significantly differentiated 5 of the arrays from the other 4 (each array was hybridized with 12 samples). Three individuals (mother-newborn) and two individuals (Red Cross) were removed since they had outlying profiles, and after exclusion of one of the technical replicates where present, 56 and 100 samples were available for analysis.

The following statistical pipeline was adopted. First, the complete profiles of 15,000 probes per sample were inter-quartile transformed (we also explored other transformations but did not observe gross distortion of the conclusions). We then performed hierarchical clustering and observed that while RNA quality was a major influence, consistent structure could be seen within the high and low quality groups, so decided to remove this effect statistically. We fit an analysis of variance model to each probe fitting expression as a function of RNA quality with four approximately equal-sized categorical levels in the mother-newborn study (RIN<5.3; 5.3<RIN<6.5; 6.5<RIN<7.7; RIN>7.6) or three categorical levels in the Red Cross study (RIN<8.0; 7.9<RIN<9; RIN>8.9), as well as the dichotomous array effect in the Red Cross study. The standardized residuals from this model were carried further for all subsequent modeling.

Subsequently, we calculated the principal components of the gene expression profiles, retaining the first 5 PC in each analysis as documented in the text. One of the outputs of the JMP Genomics expression workflow is an estimate of the proportion of specified experimental factors that is explained by each PC. This revealed that PC2_MN_ in the first study precisely corresponds to the distinction between mother and newborn, and that PC3_ RC_ in the second study is contributed in part by the distinction between inner city and suburban residents. It also showed that neither RNA quality (RIN score) nor array effects contribute to the variation in the transformed data, and the body mass index overall was a very minor component of the variation in either study.

Gene significance was evaluated with a combination of ANOVA and multiple linear regression. For the mother-newborn study, probe intensity was modeled as a function of the fixed categorical contrasts BP1A against BP1B and of Mother against Newborn, with PC3_MN_, PC4_MN_ and PC5_MN_ as covariates. For the Red Cross study, probe intensity was modeled as a function of PC1_RC_, PC2_RC_ and PC3_RC_ using the ANOVA routine in JMP Genomics in the absence of a fixed categorical effect. Axes from each study were compared simply by correlating the estimated regression slopes from these analyses for all probes.

Appendix S1 describes a series of additional analyses that were performed to assess the impact of the RNA integrity transformation on the principal component analysis. In short, instead of adjusting the transcript abundance measures for RIN, we performed two parallel analyses to control for RNA integrity (i) by only including high RNA quality samples, and (ii) by removing all probes from the analysis that had a significant RIN effect at p<0.05. Although the percent variation explained by the top 3 PC changed slightly, all were clearly conserved across the three analyses and identified the same gene sets as the major components of variation. Furthermore, we also conducted an aggressive normalization including hybridization chip as well as RIN, and this also reduced the percent variation explained by PC1_MN_, but retained it as one of the top three components of the variance with the same transcripts contributing.

### Gene Set Enrichment

Gene set enrichment analyses were performed using a combination of DAVID functional annotation (reported in Table 3), KEGG Pathway queries, Ingenuity Pathway Analysis. In each case we set thresholds for inclusion of between 500 and 800 unique genes, noting that agreement between replicate probes for the approximately 10% of genes was complete with respect to sign of effect, and almost always estimated similar magnitudes of differential expression. Bonferroni adjustments for multiple comparison testing were used to assess the DAVID pathways, whereas the KEGG analysis relied on simple gene counts. Additionally, transcription factor and miRNA binding site enrichment, along with representation of biological pathways and drug targets was performed with ToppFun in the ToppGene suite [22] and is reported in Table S4.

## Supporting Information

Appendix S1 Description of alternative analyses that demonstrate robustness of conclusions to control for RNA integrity.
(0.47 MB PDF)

Table S1 Magnitude and significance of differential expression.
(11.21 MB XLS)

Table S2 Study design; attributes of study participants discussed in the text.
(0.07 MB XLS)

Table S3 Normalized data for both experiments, as submitted to GEO.
(9.14 MB ZIP)

Table S4 ToppFun gene enrichment results.
(1.15 MB XLS)
