# Supplementary material for: Maternal Influences on the Transmission of Leukocyte Gene Expression Profiles in Population Samples from Brisbane, Australia
Source: PLoS One. 2010 Dec 31;5(12):e14479. doi: 10.1371/journal.pone.0014479 (PMC3013110; doi:10.1371/journal.pone.0014479)

APPENDIX: CONTROLLING FOR RNA INTEGRITY

Our initial analysis of the median centered, log2 transformed data revealed that RNA quality has a major influence on the expression profiles, unsurprisingly given that many of the samples had RIN values generally considered below quality for microarray analysis. This is clearly seen in the hierarchical clustering (Ward's method, with standardization) below. RIN values are to the left, with poor quality samples in blue lettering and fair or good quality in maroon lettering. Maternal samples are at the bottom, but even within the RNA quality clusters there are subgroups that further analysis indicates correspond to the BP1 profile.

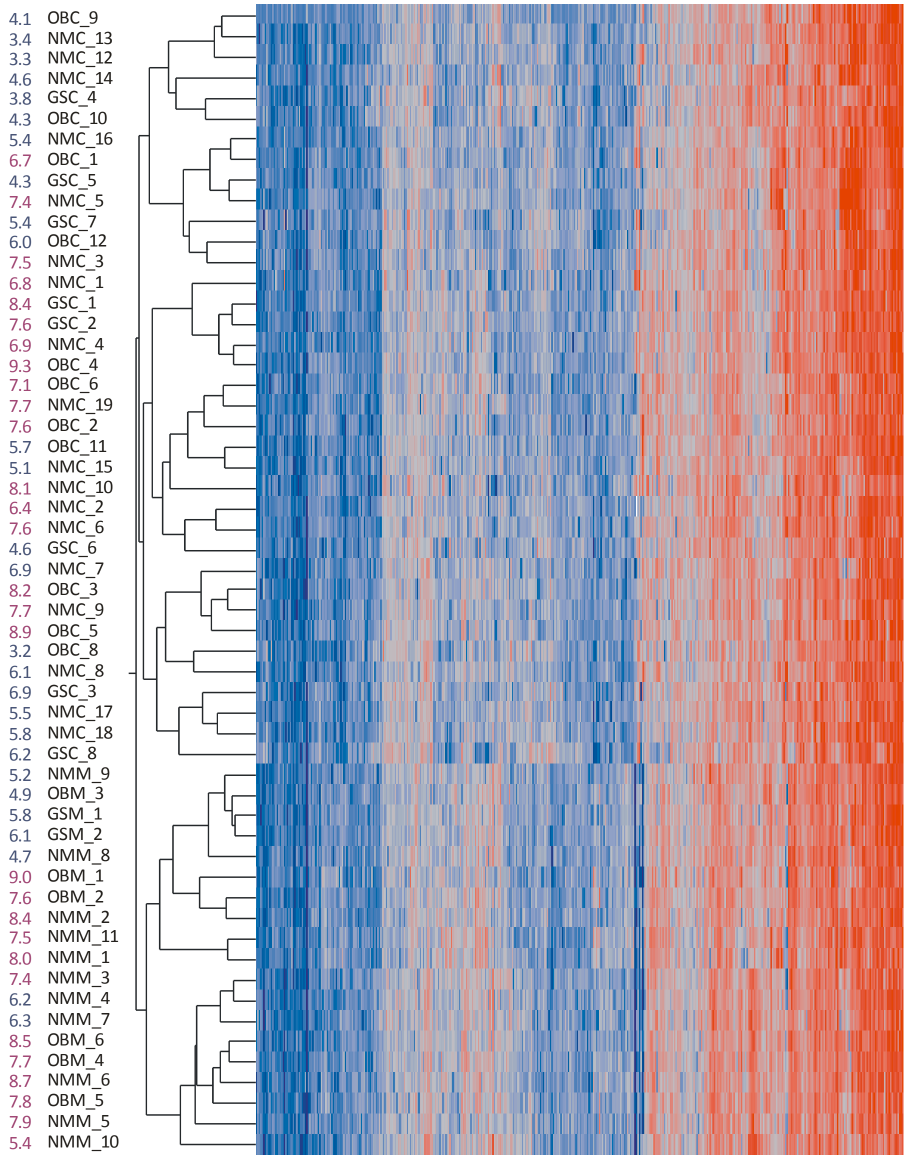

Consequently , we chose to fit the RIN effect as described in the text. Here we provide three further lines of evidence that the major profile distinguishing the BP1A and BP1B profiles is independent of the RNA quality technical issue.

1. The BP1 profile is captured by the 2nd and 3rd principal components of the original data.

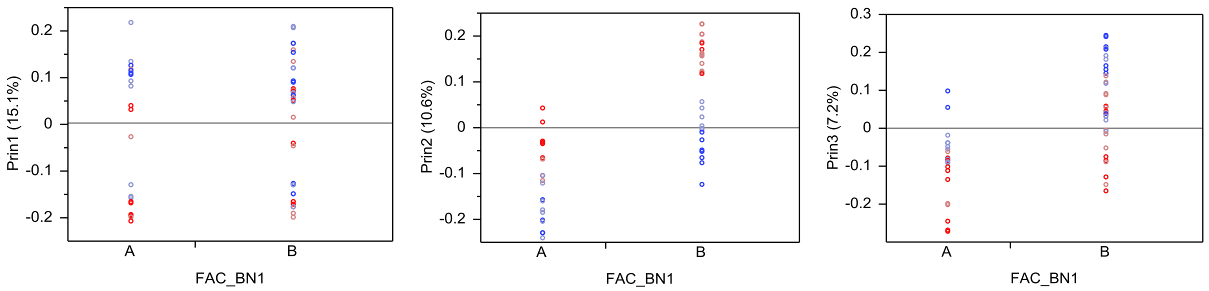

These three plots show PC1, PC2, and PC3 from the median centered, log2 values of the raw data as a function of BP1A and BP1B on the x-axis. PC1 separates mother and newborn (as seen in the hierarchical cluster heat map above, and plot to the left below). Red circles are high RNA quality (RIN>6.5) and blue circles low quality (RIN<6.5): clearly these are differentiated along both PC2 and PC3 (plots below show PC as a function of the 4 RIN levels), but BP1A and BP1B are also significantly different for both of these principal components (p<0.0001).

PC1 is Mother-Child

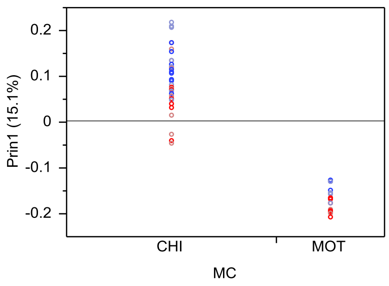

PC2 and PC3 also incorporate RIN quality

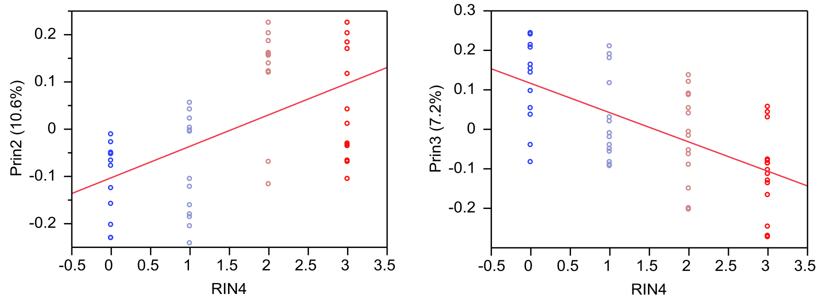

2. The BP1 profile is preserved in analysis excluding the poor RIN samples.

An analysis of the 29 samples with RIN > 6.5 (classes 2 and 3) separates BP1A (purple) and BP1B (green) along the first PC axis, and mothers (solid spots) and newborn children (circles) along the second axis. The BP assignments according to this classification are identical to the full analysis described in the text. The heat map is a symmetric clustering of pair-wise similarity of overall gene expression profiles.

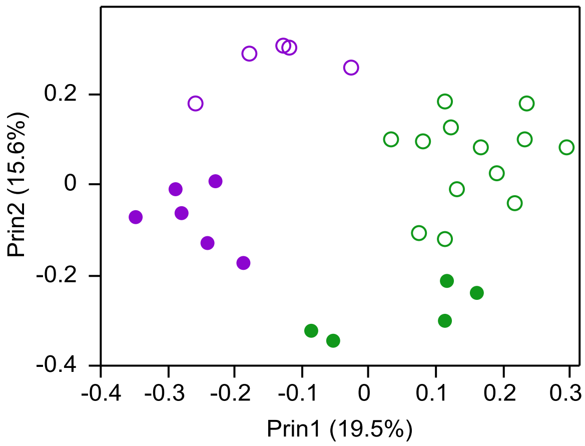

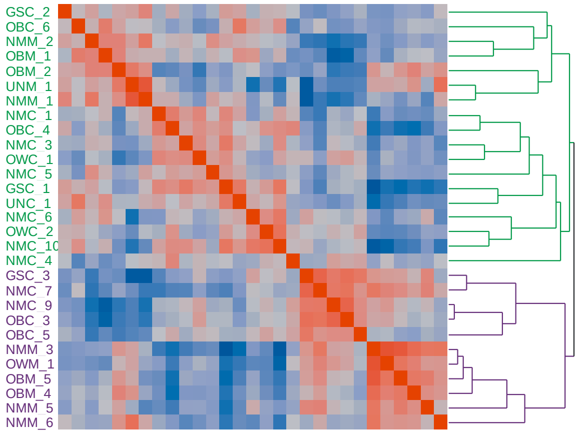

3. The BP1 profile is preserved in analysis excluding all probes with significant RIN effects.

We also tested for significance of the RIN effect in the median centered, log2 data, and identified 4,663 probes with $p<0.05$, namely approximately one third of the probes. These were removed from the dataset, leaving 10,337 probes. The data was median centered once more, and the following figures show that the two major principal axes are mother-newborn (PC1 in this case), and BP1 (PC2). Assignment of profile identity to individual samples is identical to the assignments in the full dataset after fitting the RIN effect as described in the text, and in the reduced high RIN dataset.

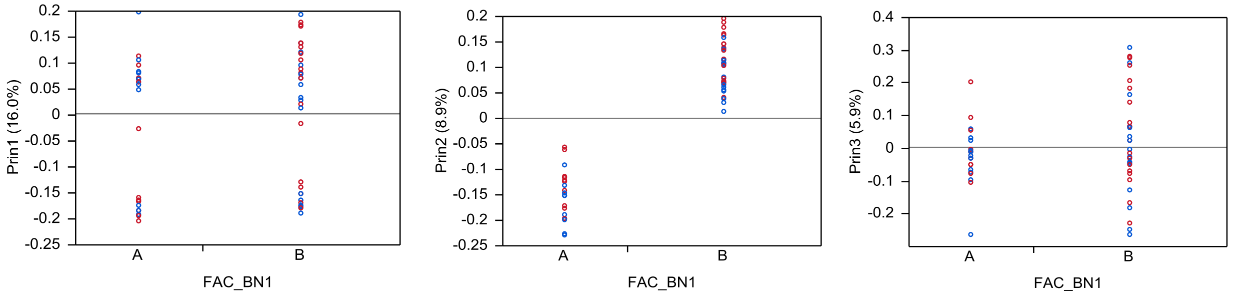

In the heat map below, clustering profiles according to overall similarity as on the previous page, RNA quality is indicated with red (high RIN) and blue (low RIN) labeling and these samples are dispersed with respect to the major clusters of gene expression profile that correspond to mother/newborn child (bottom and top respectively) and BP1A and BP1B within these groups.

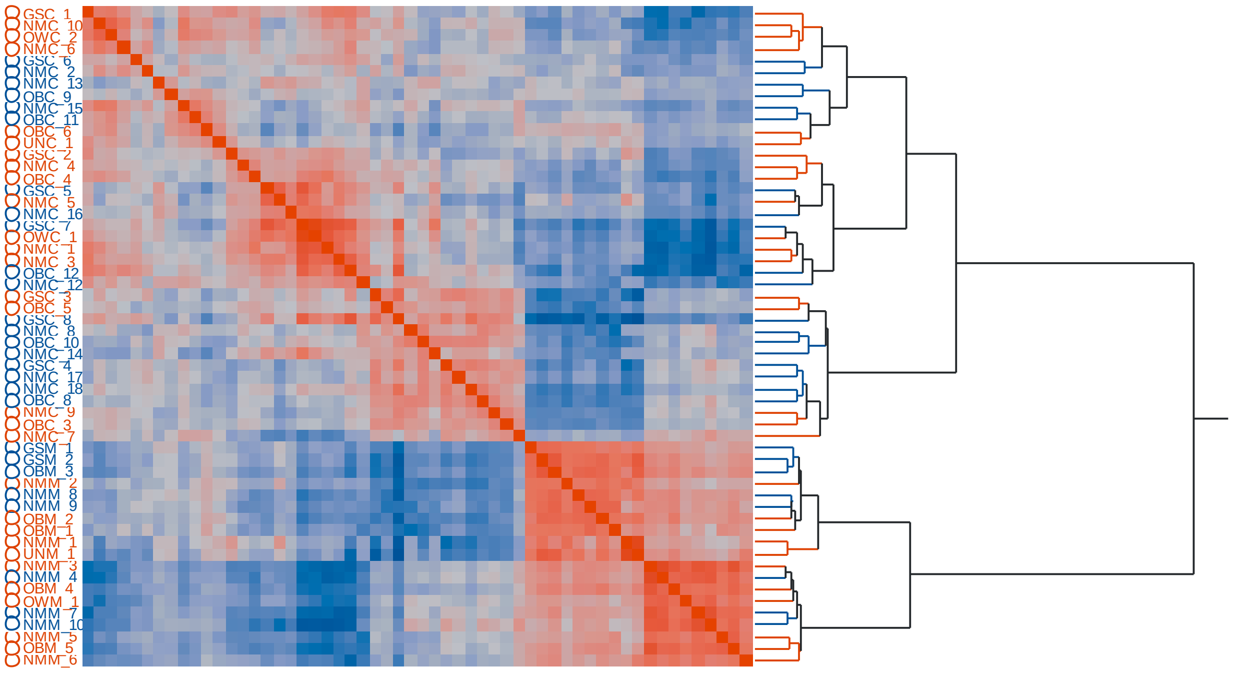

Supplement: Appendix S1 — Description of alternative analyses that demonstrate robustness of conclusions to control for RNA integrity. (0.47 MB PDF) [file pone.0014479.s001.pdf]
